# Genetic and clinical landscape of ER + /PR- breast cancer in China

**DOI:** 10.1186/s12885-023-11643-2

**Published:** 2023-12-04

**Authors:** Danian Dai, Hongmei Wu, Hongkai Zhuang, Rong Chen, Cheng Long, Bo Chen

**Affiliations:** 1 Department of Plastic and Peripheral Vascular Surgery, Guangdong Provincial People’s Hospital (Guangdong Academy of Medical Sciences), Southern Medical University, Guangzhou, 510080 Guangdong China; 2 Department of Pathology, Guangdong Provincial People’s Hospital (Guangdong Academy of Medical Sciences), Southern Medical University, Guangzhou, 510080 Guangdong China; 3 Guangdong Provincial Key Laboratory of Malignant Tumor Epigenetics and Gene Regulation, Sun Yat-Sen Memorial Hospital, Sun Yat-Sen University, Guangzhou, 510120 Guangdong China; 4 Department of Breast Oncology, State Key Laboratory of Oncology in South China, Collaborative Innovation Center for Cancer Medicine, Sun Yat-Sen University Cancer Center, Guangzhou, 510060 Guangdong China; 5 Department of Pathology, Yueyang Maternal Child Health-Care Hospital, Yueyang, 414000 Hunan China; 6 Department of Breast Cancer, Guangdong Provincial People’s Hospital (Guangdong Academy of Medical Sciences), Southern Medical University, Guangzhou, Guangdong 510080 China

**Keywords:** Breast cancer, Progesterone receptor, Estrogen receptor, NGS, MammaPrint

## Abstract

**Background:**

Estrogen receptor-positive and progesterone receptor-negative (ER + /PR-) breast cancer comprise a special type. More than 10% breast cancer patients belonged to ER + /PR-.

**Methods:**

In order to better understand this patient population, we utilized a unique dataset from China, examining the clinicopathological features and genomic profiles of ER + /PR- breast cancers. Our study involved three cohorts: Cohort 1 included 2120 unselected ER-positive female patients with re-evaluated clinicopathological and survival data; Cohort 2 comprised 442 ER-positive females who underwent genetic testing; and Cohort 3 consisted of 77 ER-positive/HER2-negative females tested with MammaPrint and BluePrint.

**Results:**

Patients were stratified into four categories based on the PR/ER ratio. Clinically, ER + /PR- tumors (PR/ER ratio = 0) showed the lowest proportion of T1 tumors (10.88%) and highest proportion of HER2-positive tumors (28.36%) than did other ER + /PR + tumors groups. The ER + /PR- group contained a higher number of underweight patients (20.20%). Independently of HER2 status, ER + /PR- patients demonstrated the poorest prognosis. Genomically, the most prevalent mutations were PIK3CA (50%) in ER + /PR + tumors and TP53 (65%) in ER + /PR- tumors. ER + /PR- tumors presented more frequent mutations in TP53, ERBB2, CDK12, SPEN, and NEB, with mutation rates of 65%, 42%, 27%, 13%, and 10%, respectively. Additionally, the Tumor Mutational Burden (TMB) was higher in the ER + /PR- group compared to the ER + /PR + group. The MammaPrint score for the ER + /PR-/HER2- group was significantly lower than that of other groups. In the BluePrint analysis, only four patients were classified as Basal-Type, all of whom were ER + /PR-/HER2-.

**Conclusions:**

In this study, we identified the clinical and genetic characteristics of ER + /PR- breast cancer patients in China. Distinct PR statuses indicated different biological processes of ER + breast cancer and survival outcomes. Future treatment strategies may need to be tailored for ER + /PR- patients.

**Supplementary Information:**

The online version contains supplementary material available at 10.1186/s12885-023-11643-2.

## Background

Steroid hormone receptors are crucial biomarkers in breast cancer, which including estrogen receptor (ER) and progesterone receptor (PR) [1]. Over 70% of breast cancers are hormone receptors positive [2]. PR serves as a biomarker for ER function, and its expression closely correlates with that of ER [3]. Mechanistically, PR is a downstream gene target of ER [4]. Since the expression activity of ER can regulate the expression of PR, ER and PR expression is generally consistent. However, inconsistent ER and PR expression also exist in some patients. Some ER-positive tumors have a partial loss or a complete lack of PR expression [5, 6].

Clinically, ER + /PR- breast cancer is still defined as Luminal subtype breast cancer, which recommends endocrine therapy. It is evident that ER + /PR- tumors have more aggressive biological and clinical characteristics compared to ER + /PR + tumors [7]. Tamoxifen is less effective against ER + /PR- tumors, so more aggressive treatments might be beneficial [8, 9]. Nevertheless, the genetic characteristics of ER + /PR + breast cancer and ER + /PR- breast cancer is not identical.

Some studies, based on western patients, have tried to reveal the characteristics of ER + PR- breast cancer during the last two decades [5, 6]. Currently, the significance of ER + PR- breast cancer remains unclear. Furthermore, differences in survival following a breast cancer diagnosis based on ethnicity and race, while considering the status of estrogen and progesterone receptors, have been observed [10]. However, we still lack a comprehensive understanding of the genetic landscape of ER + PR- breast cancer patients, particularly in Asian populations. We should not underestimate the contribution of racial disparities to breast cancer genomic traits. Therefore, it is of utmost importance to utilize data from Asian patients to explore the features of ER + /PR- breast cancer.

In this study, we included more than two thousand ER positive breast cancer patients in two large tertiary hospitals from China. We have examined ER + /PR- breast cancer patients with long-term follow-up, genomic data, the risk assessment of recurrence and intrinsic molecular subtypes data (MammaPrint and BluePrint). This study sought to enhance our understanding of the genetic and clinical characteristic underlying ER + /PR- breast cancer in China.

## Patients and methods

### Patients

This study included three cohorts. The first cohort (Cohort 1) is from Sun Yat-Sen University Cancer Centre including 2120 ER-positive unselected female patients. Those patients diagnosed of breast cancer between January 1, 2001 and December 16, 2011. Cohort 1 is comprising clinicopathological and follow-up data. Patients were followed up to April 27, 2017 or until death. The second (Cohort 2) and third cohorts (Cohort 3) are from Guangdong Provincial People's Hospital. Patients in Cohort 2 and Cohort 3 are diagnosed of breast cancer during June, 2017 to September, 2019. Cohort 2 is including 442 ER-positive female patients who had performed genetic test using a panel comprising 520 cancer-related genes. Cohort 3 is including 77 ER-positive/HER2-negative female patients who had performed MammaPrint and BluePrint test. This study was conducted in accordance with the principles of the 1964 Declaration of Helsinki. Both Sun Yat-Sen University Cancer Center Institute Research Ethics Committee (No. YB2016-002–03) and Guangdong Provincial People's Hospital Ethics Committee (No. GDREC2019497H; 2019-040H-1) approved this retrospective study. There was written informed consent from every patient enrolled.

### Immunohistochemistry (IHC)

ER and PR expression was considered positive in tumors with 1% or more positively stained nuclei. All patients are ER positive in this study. In accordance with 2013 American Society of Clinical Oncology/College of American Pathologists (ASCO-CAP) guidelines, HER2 status was evaluated. When IHC result was two-plus (2 +), HER2 status was confirmed using fluorescence in situ hybridization (FISH). Ki67 expression was measured and reported as a percentage score of positive tumor cells (range 0–100%).

### Next-Generation Sequencing (NGS)

Next generation targeted genomic DNA-sequencing of formalin-fixed paraffin-embedded tissue was performed using a panel covering 520 cancer related genes, spanning 1.64 megabases of the human genome, as previously described [11, 12]. In CLIA-certified Burning Rock Biotech (Guangzhou, China), sequencing assays were performed blinded to clinical pathological parameters.

### Tumor mutation burden (TMB) calculation

The calculation method of TMB is described in our previous research [13]. TMB per patient is determined by the ratio of non-synonymous mutations detected to the total coding region size of the panel. The mutation count encompasses non-synonymous single nucleotide variants and insertions or deletions (Indels) identified within the coding region, along with a ± 2 base pair margin upstream and downstream. This count excludes hot mutation events, copy number variations, structural variations, and germline single-nucleotide polymorphisms. Only mutations with allelic fractions equal to or exceeding 2% for tissue samples and 0.2% for plasma samples are included in the mutation count. To ensure precise TMB calculation, the maximum allelic fraction is established at 5% for tissue samples and 1% for plasma samples. The total coding region size for TMB estimation is 1.003 megabases for the 520-gene OncoScreen Plus panel.

### MammaPrint and blueprint

MammaPrint and BluePrint are based on microarray gene expression analysis. The MammaPrint test provides a definitive high-risk or low-risk assessment of tumors based on 70 gene expression signatures. BluePrint 80-gene test classified tumors into Luminal-Type, HER2-Type, or Basal-Type. Tissue samples were fixed formalin, dehydrated and embedded in paraffin. According to standard protocols, the MammaPrint and BluePrint assays were performed at the centralized Genecast Biotechnology laboratory in Beijing, China. Genecast Biotechnology has obtained the authorization of MammaPrint® and BluePrint® assays by Agendia N.V. and Agendia, Inc. All analyses were done blinded to clinical and pathological information.

### Statistical analyses

Statistical analyses were performed using SPSS version 23 and R version 3.6.2 software. Fisher's exact test or chi-square test was used when comparing these categorical variables between groups. Survival curves (Kaplan–Meier) were compared by the log-rank test. Univariate and multivariate analyses (Cox proportional hazards regression model) examined prognostic factors. Variables with *P* < 0.05 in the univariate analyses were selected for the multivariate analysis. All statistical tests were two-sided.A difference is considered significant when *P* < 0.05.

## Results

### Clinicopathological characteristics in different PR expression groups

**Fig. 1:**
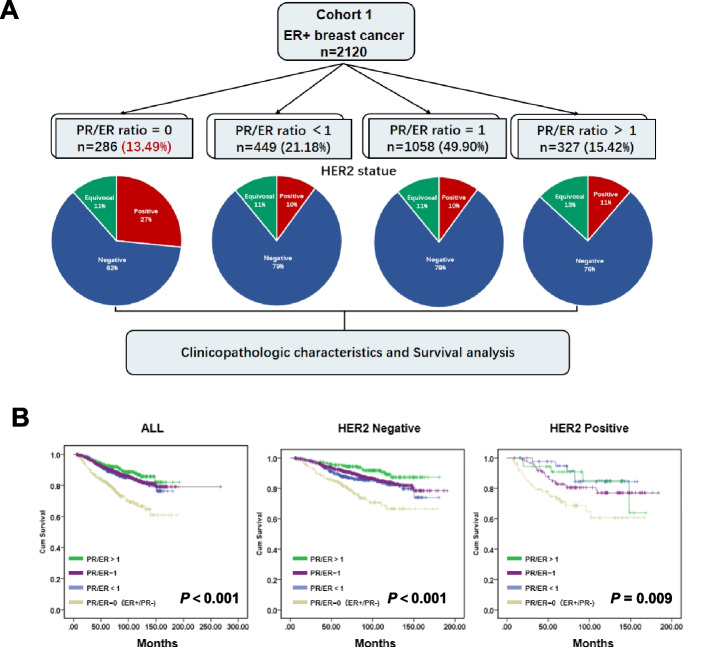
Identifying ER + /PR- breast cancer characteristics and patient survival. **A** The clinicopathologic features of ER + /PR- breast cancer from Cohort 1. The pie chart illustrates the HER2 status of patients in each group. Blue represents HER2-negative, red represents HER2-positive, and green represents HER2- equivocal. **B** Kaplan–Meier survival curves were used to assess the overall survival of patients in Cohort 1. The left panel represents the overall survival of all patients in Cohort 1. The middle panel focuses on HER2-negative patients, while the right panel presents data for HER2-positive patients

We summarize the clinicopathologic features of ER + /PR- breast cancer from Cohort 1 (Fig. 1A). Patients within Cohort 1 were stratified into four distinct groups based on their PR/ER ratio, which assesses the proportion of PR positive cells to ER positive cells: PR/ER ratio = 0 (indicating PR negative), PR/ER ratio < 1, PR/ER ratio = 1, and PR/ER ratio > 1 (Fig. 1A). Among the patients in this cohort, 286 individuals were assigned to the PR/ER ratio = 0 group, representing 13.49% of the total ER + patients. We conducted a thorough comparison of baseline characteristics across these groups, as detailed in Table 1. It was observed that, in contrast to both the PR/ER ratio = 1 and PR/ER ratio > 1 groups, the PR/ER ratio = 0 and PR/ER ratio < 1 groups displayed a higher incidence of tumors in individuals aged 50 years or older (*P* < 0.001). Moreover, a statistically significant difference in BMI was evident among the four groups, with a notable prevalence of underweight patients in the PR/ER ratio = 0 group (20.20%) (*P* = 0.018). Furthermore, the PR/ER ratio = 0 group exhibited the lowest proportion of T1 tumors (10.88%) (*P* = 0.034) and the highest proportion of HER2-positive tumors (28.36%) (*P* < 0.001). In contrast, no significant variations were observed among the four groups concerning Blood Type, Grade, LN status, M stage, and TNM Stage (Table 1).

**Table 1:** Clinicopathological characteristics of breast cancer patients from Cohort 1

	**(ER + /PR- GROUP)**		**(ER + /PR + GROUP)**						***P*-value**
	**PR/ER = 0**		**PR/ER < 1**		**PR/ER = 1**		**PR/ER > 1**		
	n	%	n	%	n	%	n	%	
**Age**									** < 0.001**
< 50	119	9.38%	206	16.25%	687	54.18%	256	20.19%	
≥ 50	167	19.60%	243	28.52%	371	43.54%	71	8.33%	
**BMI**									**0.018**
Underweight	20	20.20%	12	12.12%	50	50.51%	17	17.17%	
Normal weight	140	11.82%	241	20.35%	603	50.93%	200	16.89%	
Overweight	112	14.93%	176	23.47%	360	48.00%	102	13.60%	
UNKNOWN	14	16.09%	20	22.99%	45	51.72%	8	9.20%	
**Blood Type**									0.367
A	83	13.47%	135	21.92%	305	49.51%	93	15.10%	
B	70	13.21%	128	24.15%	244	46.04%	88	16.60%	
O	109	13.73%	155	19.52%	407	51.26%	123	15.49%	
AB	12	10.08%	22	18.49%	70	58.82%	15	12.61%	
UNKNOWN	12	19.67%	9	14.75%	32	52.46%	8	13.11%	
**Grade**									0.227
1	25	10.82%	40	17.32%	130	56.28%	36	15.58%	
2	163	12.95%	280	22.24%	623	49.48%	193	15.33%	
3	98	15.56%	129	20.48%	305	48.41%	98	15.56%	
**T stage**									**0.034**
T1	72	10.88%	139	21.00%	350	52.87%	101	15.26%	
T2	167	14.13%	250	21.15%	567	47.97%	198	16.75%	
T3	30	18.99%	32	20.25%	84	53.16%	12	7.59%	
T4	17	14.41%	28	23.73%	57	48.31%	16	13.56%	
**LN status**									0.052
Positive	120	11.47%	233	22.28%	531	50.76%	162	15.49%	
Negative	166	15.46%	216	20.11%	527	49.07%	165	15.36%	
**M stage**									0.362
M0	278	13.35%	439	21.09%	1042	50.05%	323	15.51%	
M1	8	21.05%	10	26.32%	16	42.11%	4	10.53%	
**TNM Stage**									0.09
I-II	204	12.90%	321	20.29%	803	50.76%	254	16.06%	
III-IV	82	15.24%	128	23.79%	255	47.40%	73	13.57%	
**HER2 status**									** < 0.001**
Positive	76	28.36%	45	16.79%	110	41.04%	37	13.81%	
Negative	177	10.98%	355	22.02%	833	51.67%	247	15.32%	
Equivocal	33	13.75%	49	20.42%	115	47.92%	43	17.92%	

### Survival analysis results and subgroup analysis

The median follow-up time for the 2120 enrolled patients was 81.8 months, with a range of 5.1 to 192.8 months (censored). The estimated mean overall survival (OS) varied as follows: 141.0 months (95% CI, 131.4–150.6) for patients with a PR/ER ratio = 0, 157.1 months (95% CI, 151.4–162.7) for those with a PR/ER ratio < 1, 166.2 months (95% CI, 162.4–167.0) for those with a PR/ER ratio = 1, and 172.8 months (95% CI, 166.2–179.4) for those with a PR/ER ratio > 1. Notably, patients in the PR/ER ratio = 0 group exhibited the lowest OS, followed by the other three groups (*P* < 0.001) (Fig. 1B left). Subgroup analyses were conducted based on HER2 expression status, excluding patients with uncertain HER2 expression status. These subgroup analyses consistently revealed that patients in the PR/ER ratio = 0 group had the poorest OS, irrespective of their HER2 expression status (Fig. 1B middle and right).

### Mutation landscape of ER-positive breast cancer with different PR statue

**Fig. 2:**
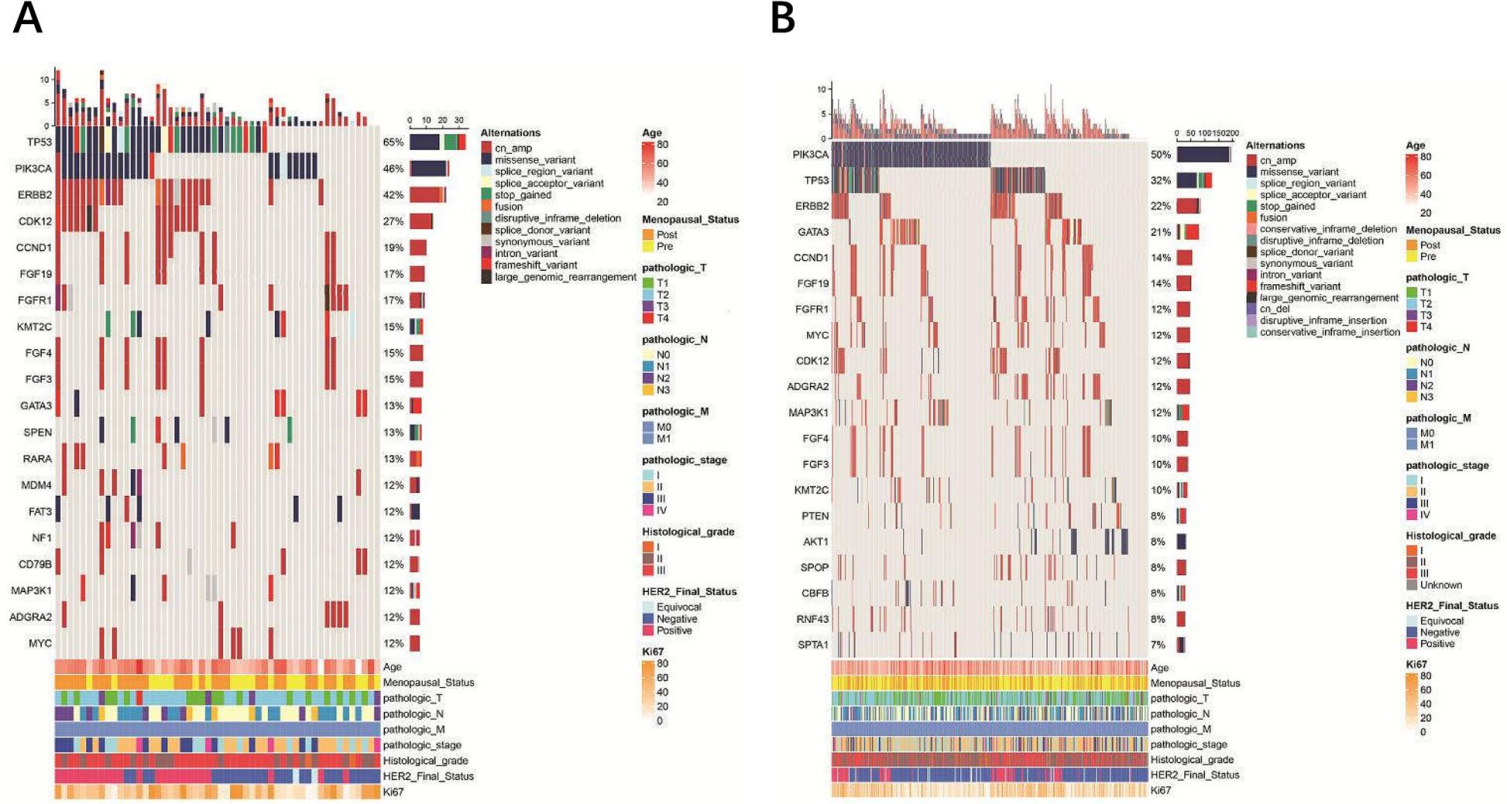
A summary of the genomic characteristics of 442 ER-positive female patients. The mutation landscape of the (**A**) ER + /PR- group (*n* = 52) and (**B**) ER + /PR + group (*n* = 390) are depicted (only shown top 20 genes). The sidebar provides a summary of the percentage of tumors with mutations in each gene. Different colors are used to distinguish between different types of mutations. Clinicopathological features are annotated at the bottom of the figure. "cn_amp" for copy number amplification, and "cn_del" for copy number deletion

**Fig. 3:**
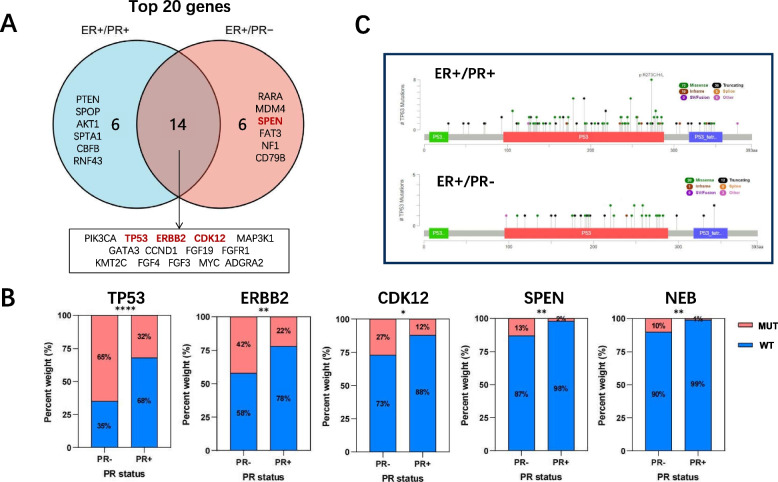
Genetic differences and TP53 mutation patterns in ER + /PR- and ER + /PR + tumors. **A** A Venn diagram was used to identify mutation overlap between the ER + /PR- group and ER + /PR + group. Genes that are differentially mutated between the two groups (*P* < 0.05) are highlighted in red. **B** The differentially mutated genes (TP53, ERBB2, CDK12, SPEN and NEB) between the ER + /PR- group and ER + /PR + group. Significance levels are indicated as follows: * for *P* < 0.05, ** for *P* < 0.01, and **** for *P* < 0.0001. **C** Lollipop plot showing mutations in the TP53 gene. The upper section represents the ER + /PR + group, while the lower section represents the ER + /PR- group. Different colors are used to distinguish between various mutation types

We performed NGS sequencing of a 520-gene panel on 442 ER-positive female patients in Cohort 2 (The characteristics of patients in Supplemental Table S1). Based on the results of IHC and FISH testing, the HER2 status of this group of patients is as follows: 102 cases are HER2-positive, 316 cases are HER2-negative, and 24 cases are HER2-equivocal. A total of 4,160 mutations were identified across 409 genes, with 715 mutations in the ER + /PR- group (*n* = 52) and 3,445 mutations in the ER + /PR + group(*n* = 390). The distribution of mutation types in ER + /PR- and ER + /PR + patients is similar (see Supplemental Table S2 and Supplemental Table S3). Utilizing previously identified driver mutations as a basis [14], we defined somatic driver base substitutions and indel mutations. We then searched for driver mutations meeting these criteria in both the ER + /PR- and ER + /PR + groups. Within the ER + /PR- group, we identified 37 probable driver mutations of this category, whereas in the ER + /PR + group, we found 296. Subsequently, we examined the impact of incorporating recurrent copy number changes as driver mutations. In the ER + /PR- group, we detected 50 copy number changes in genes such as CCND1, ERBB2, KRAS, CCNE1, MYC, MDM2, PIK3CA, and ESR1, all classified as driver mutations. Meanwhile, in the ER + /PR + group, we identified 220 copy number changes affecting 12 genes, including MYC, ERBB2, CCND1, MDM2, FGFR2, IGF1R, ESR1, AKT1, PIK3CA, EGFR, KRAS, and CCNE1, which were classified as driver mutations. The mutation landscapes of the ER + /PR- group (Fig. 2A) and the ER + /PR + group (Fig. 2B) are presented (only shown top 20 genes). In ER + /PR- tumors, TP53 exhibited the highest mutation frequency (65%), whereas in ER + /PR + tumors, PIK3CA had the highest mutation frequency (50%). A Venn diagram was used to identify top 20 genes of mutation overlap between the two groups (Fig. 3A). To further understand the differences in mutated genes between ER + /PR- and ER + /PR + tumors, we conducted an analysis. Five genes were found to be differentially mutated between the two groups, as illustrated in Fig. 3B. ER + /PR- tumors exhibited a higher incidence of TP53, ERBB2, CDK12, SPEN, and NEB variants (with mutation rates of 65%, 42%, 27%, 13%, and 10%, respectively). As TP53 was not only the most frequently mutated gene but also differentially mutated, we conducted a more in-depth investigation into the TP53 mutation spectrum in the two groups (Fig. 3C). In this analysis, a hotspot in codon 273 was identified in the ER + /PR + group (R273C/H/L, n = 8, where 'n' represents the number of mutations), while no mutations in codon 273 were observed in the ER + /PR- group. In the ER + /PR- group, there doesn't seem to be any obvious hotspot regions for TP53 mutations. Apart from R248W, R342*, and Y220C, each having 2 mutations, mutations at other sites occurred only once.

### Tumor mutation burden in ER + /PR- group and ER + /PR + group

**Fig. 4:**
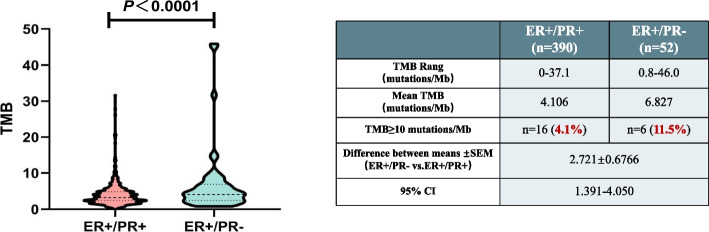
Tumor mutation burden comparison between ER + /PR- and ER + /PR + groups. ER + /PR- group displayed significantly higher TMB (6.827 muts/Mb) than ER + /PR + (4.106 muts/Mb). TMB > 10 muts/Mb was classified as high TMB. In ER + /PR + group, 4.1% had high TMB, while 11.5% in ER + /PR- group exhibited elevated TMB

A pivotal aspect of our study was the examination of TMB in the ER + /PR- and ER + /PR + groups. The results showed that the TMB in the ER + /PR- group significantly surpassing that of the ER + /PR + group (*P* < 0.0001) (Fig. 4). We calculated the mean mutation burden for tumors in the entire study population. In the ER + /PR + group, the mean TMB was 4.106, with TMB values ranging from 0 to 37.1 muts/Mb. In contrast, the ER + /PR- group exhibited a significantly higher mean TMB of 6.827, with a broader range of TMB values spanning from 0.8 to 46.0 muts/Mb. These findings shed light on a distinctive mutational pattern within ER + /PR- breast cancer, which may carry significant clinical implications for prognosis and treatment strategies. Subsequently, we applied a standardized criterion to define high TMB, classifying TMB values exceeding 10 muts/Mb as indicative of a high TMB status [15]. This definition allowed us to identify patients with an exceptionally high mutation burden. Notably, within the ER + /PR + group, 4.1% of the individuals exhibited high TMB, while within the ER + /PR- group, a substantial 11.5% of patients displayed elevated TMB levels. These observations underscore the substantial divergence in mutation profiles between these two groups, thus emphasizing the potential impact of PR status on the genetic landscape of breast cancer.

### Recurrence risk and intrinsic molecular subtypes of different PR statue groups

**Fig. 5:**
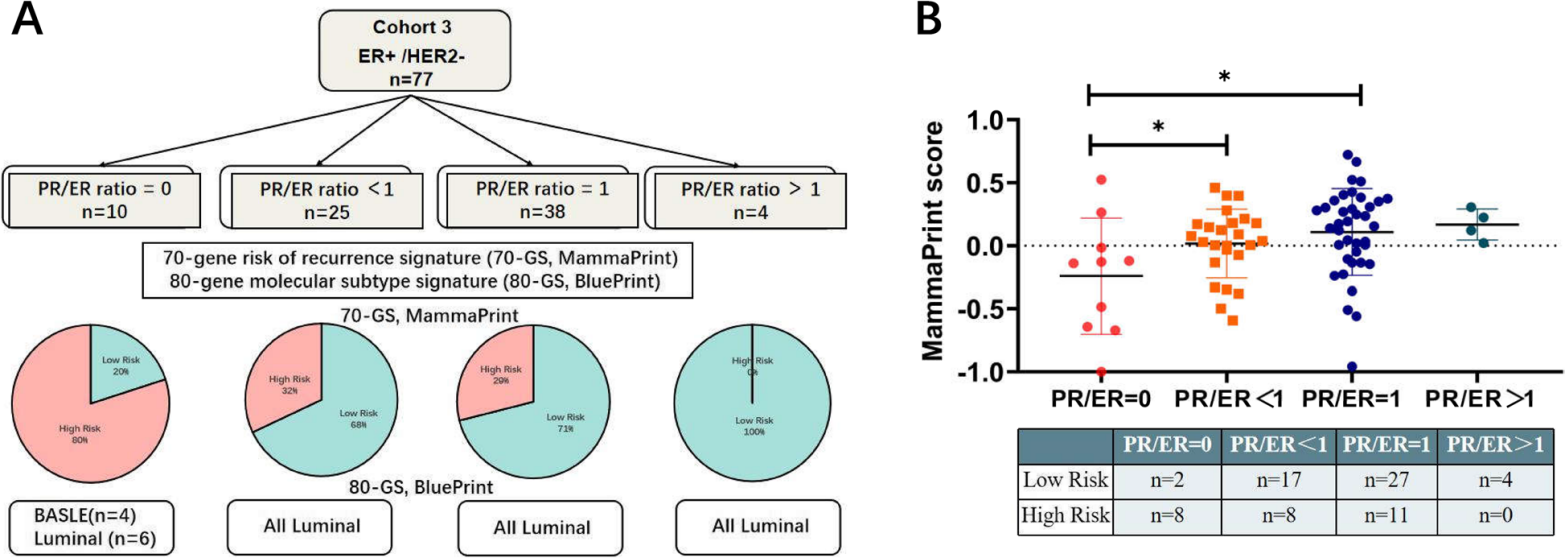
Recurrence risk and intrinsic molecular subtypes of different PR statue groups. **A** MammaPrint and BluePrint were performed in 77 ER + /HER2- patients (Cohort 3). The pie chart represents the risk stratification of MammaPrint. Red represents high risk, while green represents low risk. **B** The MammaPrint score in 77 ER + /HER2- patients. The grouped scatter plot illustrates the MammaPrint scores of patients. The number of high-risk and low-risk patients in each group is listed in the table below the scatter plot

MammaPrint is a risk prediction model which can be applied to ER + /HER2- patients with negative lymph nodes or 1–3 positive lymph nodes. Therefore, in our study MammaPrint was performed only in these 77 ER + /HER2- patients, and HER2 + patients were excluded. In this cohort, we also separated patients into four groups based on their PR/ER ratios (PR/ER ratio = 0, *n* = 10; PR/ER ratio < 1, *n* = 25; PR/ER ratio = 1,*n* = 38 and PR/ER ratio > 1,*n* = 4). The MammaPrint results (Fig. 5A) revealed that within the PR/ER ratio = 0 group, 80% of patients (*n* = 8) exhibited a high risk of distant recurrence, while the remaining 20% (*n* = 2) demonstrated a low risk. Notably, the proportion of high-risk patients in the PR/ER ratio = 0 group was significantly higher in comparison to the other groups. The distribution of high-risk patients stood at 32% in the PR/ER ratio < 1 group, 29% in the PR/ER ratio = 1 group, and 0% in the PR/ER ratio > 1 group. Furthermore, the MammaPrint score of the PR/ER ratio = 0 group was markedly lower than that of the other groups (Fig. 5B). Due to the small number of patients in the PR/ER ratio > 1 group (only 4 patients), statistical differences could not be established in this particular group. Subsequently, we performed a BluePrint analysis on the same cohort of 77 patients (Fig. 5A). The results of this analysis indicated that 73 patients were classified as Luminal-Type, while only 4 patients were categorized as Basal-Type. Importantly, all four Basal-Type patients belonged to the PR/ER ratio = 0 group.

## Discussion

Exploring tumor heterogeneity is crucial for understanding tumor evolution and providing clinicians with guidelines for developing tailored therapeutic regimens. The majority of breast cancers are ER-positive, and the primary treatment for these patients is hormonal therapy [16]. However, a subset of ER-positive breast cancer patients still experiences recurrences despite undergoing endocrine therapy [17]. Two crucial molecules for evaluating breast cancer heterogeneity and the advantages of hormonal therapy are the steroid hormone receptor ER and the progesterone receptor PR. This study focuses on the clinicopathological features and genomic changes in ER + /PR- breast cancers, utilizing an exclusive patient dataset from China, which includes comprehensively annotated clinical data, survival follow-up, and genomic information.

Even though this study is retrospective, it does comprise an unselected breast cancer population without exclusions or selection biases. In ER + patients (Cohort 1), ER + /PR- patients comprise approximately 13.49% of the total. This result is similar to that reported by SEER database (15.35%) [1]. ER + /PR- breast cancers are more frequently observed in older women and underweight patients, and this group had the lowest proportion of T1 tumors and the highest proportion of HER2-positive tumors. Consistent with previous research, our study confirms that ER + /PR- tumors display more aggressive characteristics and higher HER2 expression compared to ER + /PR + tumors. The absence of PR expression may reflect hyperactive cross talk between growth factor signaling pathways and ER [3]. Previous studies have also explored whether PR expression serves as an independent prognostic variable. A European study found that the prognostic effect of PR-negativity in the ER + /HER2- group becomes most pronounced beyond 6 years of follow up [18]. Bae et al. observed that ER + /PR-/HER2- tumors were associated with worse survival outcomes than ER + /PR + /HER2- tumors, though PR negativity was not a significant prognostic factor in tumors with HER2 overexpression [6]. A SEER database study found that ER + /PR- breast cancer has a prognosis midway between that of the ER + /PR + and ER-/PR- subtypes [1]. In our ER-positive series, regardless of HER2 status, ER + /PR- patients exhibit the poorest prognosis. This phenomenon could be linked to variations in race and healthcare services across different regions. Additional studies are required to prospectively confirm these findings.

Our research has unveiled somatic mutations in ER-positive breast cancers. The most prevalent mutations in ER + /PR + tumors and ER + /PR- tumors are PIK3CA (50%) and TP53 (65%), respectively. These findings align with previous studies that have identified TP53 and PIK3CA mutations as common in breast cancer [19]. In ER + /PR- breast cancer, tumor activation of non-canonical ER-signaling leads to increased activation of the PI3K and MAPK pathways at the cellular level [20]. Furthermore, our study reveals that ER + /PR- breast tumors exhibit a higher incidence of variants in TP53, ERBB2, CDK12, SPEN, and NEB, with variant rates of 65%, 42%, 27%, 13%, and 10%, respectively. based on the TCGA dataset discovered that ER + /PR-/HER2- tumors have higher TP53 mutation rates and lower PIK3CA mutation rates compared to ER + /PR + HER2- tumors, along with a higher frequency of ZNF703 and RPS6KB1 amplification events [21]. TP53 is a tumor suppressor gene located on chromosome 17p13.1 and is frequently inactivated by mutations or deletions. Ahn et al. analyzed mutational of exons 5–9 of the TP53 in ER-positive breast cancer by PCR amplification and direct sequencing. Since they did not utilize NGS, they identified somatic TP53 mutations in only 10.3% of ER-positive tumors. But, similar to our findings, they concluded that the TP53 mutation rate was significantly higher in ER + /PR- tumors compared to ER + /PR + tumors (*P* = 0.039) [22]. TP53 mutations are associated with primary endocrine resistance in breast cancer [23]. Codons 273 is a hotspot for TP53 mutations found in most human cancers, including breast cancer [24]. In our study, despite the high overall TP53 mutation rate, no mutation in codon 273 was observed in the ER + /PR- group. This characteristic may impact the sensitivity of therapy in ER + /PR- patients. Previous esearch has shown that breast cancer patients with codon 273 mutations are more sensitive to chemotherapy compared with other TP53 mutant patients and TP53 wild-type patients [25].

Our investigation revealed a higher proportion of HER2-positive tumors in ER + /PR- patients. Therefore, it is reasonable that ER + /PR- patients have more ERBB2 gene amplified than other group. CDK12 is located approximately 200 kb proximal to the ERBB2 gene [26]. In breast cancer, CDK12 frequently displays co-amplification and cooperation with the ERBB2 and interaction with oncogenic pathways, such as IRS1-ErbB-PI3K signaling [27]. SPEN is recognized as a tumor-suppressor gene, and its deletion or intragenic mutation may contribute to breast cancer progression [28]. SPEN binds ERα and exerts a negative regulatory influence on the transcription of Erα target genes. It is a candidate predictive biomarker of tamoxifen response [29]. The functional roles of NEB in breast cancer have been poorly studied. In breast cancer, the median TMB significantly varies depending on the tumor subtype, with HR-/HER2- tumors exhibiting the highest TMB, followed by HER2 + and HR + /HER2- tumors [30]. Our study also observed differences in TMB between the ER + /PR- and ER + /PR + groups, with the ER + /PR- group displaying a higher TMB. Moreover, ER + /PR- tumors had a higher percentage of cases with high TMB. High TMB is indicative of genomic instability and an abundance of tumor neoantigens [31]. In a study by Xie et al. using the METABRIC cohort, five distinct immune subtypes were identified within ER + /PR-/HER2- breast cancer [32]. This suggests potential variations in immunogenicity between the two groups, warranting further investigation.

Recently, Zheng et al. reported that patients with ER + /PR − Ki67high tumors were more likely to have high recurrence scores to receive adjuvant chemotherapy [4]. By PAM50 genomic assay, about 20% ER + /PR-/HER2- tumors were defined as non-luminal-like subgroup and enriched biosynthesis, metabolism and DNA replication pathways [21]. In our study, we conducted MammaPrint and BluePrint analyses on a cohort of 77 ER + /HER2- patients.. The results revealed that 80% of ER + /PR-/HER2- patients (*n* = 8) had a high-risk profile. Among these 77 patients, only four patients were Basal-Type, and interestingly, all of them belonged to the ER + /PR-/HER2- subgroup. Our findings confirmed the genetic heterogeneity of ER + /PR-/HER2- tumors, and proved that the genetic characteristics of ER + /PR-/HER2- tumors are more malignant. Here, we note several limitations to our work. Firstly, due to the retrospective of this study, inherent biases may be present. Notably, detailed patient information regarding chemotherapy and radiation therapy is unavailable. Secondly, some patients are missing the FISH information of HER2 and cannot judge the final status of HER2. Thirdly, patients in Cohort 2 and Cohort 3 have not been followed up for a long time, so survival analysis cannot be carried out to link their genetic characteristics with prognosis in the future, an external validation cohort is required for our study to make the results more compelling. Fourthly, while our findings provide valuable insights into the genetic landscape of ER + /PR- breast cancer, we did not extensively investigate the mechanistic underpinnings of these observed variations. Understanding these mechanisms is pivotal for a comprehensive grasp of the disease and the development of targeted treatment strategies.

## Conclusions

In this large retrospective study, we identified the clinical and genetic characteristics of ER + /PR- breast cancer patients in China. Distinct PR statuses indicated different biological processes of ER + breast cancer and survival outcomes. ER + /PR- patients might require different treatment strategies in the future.

### Supplementary Information

**Additional file 1: Supplemental Table S1.** Baseline characteristics of patients in Cohort 2.

**Additional file 2: Supplemental Table S2.** Mutations in the ER+/PR- group.

**Additional file 3: Supplemental Table S3.** Mutations in the ER+/PR+ group.

## Data Availability

All data can be viewed in the National Omics Data Encyclopedia (NODE; http://www.biosino.org/node) by pasting the accession OEP001295 (http://www.biosino.org/node/project/detail/OEP001295) and OEP001992(http://www.biosino.org/node/project/detail/OEP001992). Data information available from the corresponding author on reasonable request in accordance with Chinese law for genomic data.

## Abbreviations

ASCO-CAP: American Society of Clinical Oncology/College of American Pathologists
CDK12: Cyclin-dependent kinase 12
DNA: Deoxyribonucleic Acid
ER: Estrogen Receptor
ERBB2: Human Epidermal Growth Factor Receptor 2
FISH: Fluorescence in Situ Hybridization
HER2: Human Epidermal Growth Factor Receptor 2
HR: Hormone Receptor
IHC: Immunohistochemistry
MAPK: Mitogen-Activated Protein Kinase
METABRIC: Molecular Taxonomy of Breast Cancer International Consortium
NEB: Next-Generation Sequencing
OS: Overall Survival
PIK3CA: Phosphatidylinositol-4,5-Bisphosphate 3-Kinase, Catalytic Subunit Alpha
PR: Progesterone Receptor
RPS6KB1: Ribosomal Protein S6 Kinase B1
SPEN: Split Ends
TCGA: The Cancer Genome Atlas
TP53: Tumor Protein 53
TMB: Tumor Mutation Burden
ZNF703: Zinc Finger Protein 703
